# Metabolic requirements of human pro-inflammatory B cells in aging and obesity

**DOI:** 10.1371/journal.pone.0219545

**Published:** 2019-07-09

**Authors:** Daniela Frasca, Alain Diaz, Maria Romero, Seth Thaller, Bonnie B. Blomberg

**Affiliations:** 1 Department of Microbiology and Immunology, University of Miami Miller School of Medicine, Miami, FL, United States of America; 2 Department of Surgery, Division of Plastic and Reconstructive Surgery, University of Miami Miller School of Medicine, Miami, FL, United States of America; 3 Sylvester Comprehensive Cancer Center, University of Miami Miller School of Medicine, Miami, FL, United States of America; Université catholique de Louvain, BELGIUM

## Abstract

The subset of pro-inflammatory B cells, called late memory, tissue-like or double negative (DN), accumulates in the blood of elderly individuals. Here we show that DN B cells do not proliferate and do not make antibodies to influenza antigens, but they secrete antibodies with autoimmune reactivity, in agreement with their membrane phenotype (CD95+CD21-CD11c+) and their spontaneous expression of the transcription factor T-bet. These cells also increase in the blood of individuals with obesity and autoimmune diseases, but causative mechanisms and signaling pathways involved are known only in part. In the present paper we compare frequencies and metabolic requirements of these cells in the blood of healthy individuals of different ages and in the blood and the subcutaneous adipose tissue (SAT) of individuals with obesity. Results show that DN B cells from young individuals have minimal metabolic requirements, DN B cells from elderly and obese individuals utilize higher amounts of glucose to perform autoimmune antibody production and enroll in aerobic glycolysis to support their function. DN B cells from the SAT have the highest metabolic requirements as they activate oxidative phosphorylation, aerobic glycolysis and fatty acid oxidation. DN B cells from the SAT also show the highest levels of ROS and the highest levels of phosphorylated AMPK (5’-AMP activated kinase) and Sestrin 1, both able to mitigate stress and cell death. This metabolic advantage drives DN B cell survival and function (secretion of autoimmune antibodies).

## Introduction

Aging is associated with poor B cell function and decreased production of protective antibodies and we have shown that both systemic and B cell intrinsic inflammation contribute to this [1–3]. Aging is also associated with increased production of autoimmune antibodies. Aging is characterized by increased low-grade chronic inflammation, called “inflammaging”, which is a risk factor for morbidity and mortality of elderly individuals as it is implicated in the pathogenesis of several disabling diseases, including type-2 diabetes mellitus [4], osteoporosis [5], Alzheimer’s disease [6], rheumatoid arthritis [7], and coronary heart disease [8]. Several factors contribute to inflammaging, including polymorphisms in the promoter regions of pro-inflammatory genes, chronic stimulation of immune cells with viruses, changes in the gut microbiome, increased permeability of the intestine, and engagement of innate receptors by endogeneous signals such as damage-associated molecular patterns, as reviewed in [9]. Cellular senescence is also a significantly contributor to inflammaging, due to the acquisition of the senescence-associated secretory phenotype (SASP) by immune cells [10], fibroblasts [11, 12] and endothelial cells [13]. This phenotype is characterized by increased secretion of pro-inflammatory molecules (cytokines, chemokines, micro-RNAs), growth factors and proteases [14].

We have recently shown that markers of the SASP are highly expressed in B lymphocytes from elderly individuals. We found that only memory B cells express SASP markers, and especially the CD19+IgD-CD27- B cell subset, called late memory (LM), tissuelike or double negative (DN), which is the most pro-inflammatory B cell subset, as compared to IgM memory and switched memory B cells [15]. This subset, that we previously called LM [15] and now DN in agreement with the other groups, has been reported to be increased in the blood of healthy elderly individuals [15, 16], and in patients with autoimmune [17–22] and infectious diseases [23–25]. These results suggest that these cells may expand *in vivo* in the presence of autoantigens or pathogen-derived antigens, in the context of a favorable inflammatory microenvironment, leading to the production of pathogenic (autoimmune) or protective antibodies, respectively. DN B cells are transcriptionally active and affect the microenvironment by secreting pro-inflammatory mediators which in turn sustain and propagate the inflammatory response. Expression of SASP markers in DN B cells is associated with activation of NF-kB, due to spontaneous activation of AMP-activated protein kinase (AMPK), the energy sensing enzyme and key metabolic regulator ubiquitously expressed in mammalian cells [26]. Only DN B cells show spontaneous activation of AMPK, suggesting that senescence and signaling pathways sensing nutrients (i.e. glucose) converge to regulate functional responses in these cells [15], similar to pro-inflammatory T [27, 28] and NK [29] cell subsets.

To date, published studies in humans have only shown the accumulation of DN B cells with age, obesity, autoimmunity or infections, but causative mechanisms and signaling pathways involved are known only in part. In the present study, we compare DN and naïve B cells (the most frequent B cell subset in blood able to undergo in vivo and in vitro immunoglobulin class switch), and we show that DN B cells do not proliferate and do not secrete antibodies against influenza antigens but they have autoimmune reactivity. Moreover, we compare frequencies, function and metabolic requirements of DN cells in the peripheral blood of healthy individuals of different ages, in the blood of individuals with obesity and in the subcutaneous adipose tissue (SAT) of individuals with obesity undergoing weight reduction surgeries. The role of metabolic changes in driving the secretion of pathogenic (autoimmune) antibodies is unknown.

## Materials and methods

### Subjects

Experiments were performed using blood isolated from young (n = 20 individuals, 25–55 years) and elderly (n = 20 individuals, ≥65 years) individuals, as well as from the blood of young individuals with obesity (n = 20 individuals, 25–55 years). We also recruited in the study females undergoing breast reduction surgery at the Division of Plastic and Reconstructive Surgery at the University of Miami Hospital [n = 22, 25–50 years, Body Mass Index (BMI, kg/m^2^) 31–49]. The individuals participating in the study were all healthy, screened for diseases known to alter the immune response or for consumption of medications that could alter the immune response. We excluded subjects with Diabetes, Congestive Heart Failure, Cardiovascular Disease, Chronic Renal Failure, malignancies, renal or hepatic diseases, autoimmune diseases, infectious disease, trauma or surgery, pregnancy, or documented current substance and/or alcohol abuse.

Study participants provided written informed consent. The study was reviewed and approved by Institutional Review Board (IRB, protocols #20070481 and #20160542), which reviews all human research conducted under the auspices of the University of Miami.

### Flow cytometry

PBMC (1x10^6^ cells) or 5x10^5^ cells from the Stromal Vascular Fraction (SVF) of the SAT were stained for 20 min at room temperature with the following antibodies: Live/Dead detection kit (InVitrogen 1878898), anti-CD45 (Biolegend 368540), anti-CD19 (BD 555415), anti-CD27 (BD 555441) and anti-IgD (BD 555778) to measure naive (IgD+CD27-), IgM memory (IgD+CD27+), switched memory (IgD-CD27+), and DN (IgD-CD27-) B cells. To measure membrane expression of markers associated with autoimmunity, B cells were also stained with anti-CD95 (Biolegend 305635), anti-CD21 (Biolegend 354911), anti-CD11c (Biolegend 301625) antibodies. To measure intracellular (ic) levels of phosphorylated STAT3, phosphorylated AMPK and total AMPK, cells were fixed and permeabilized with PBS containing 0.2% Tween and then stained for additional 20 min at room temperature with anti-phosho-STAT3 antibody (Tyr 705, #4113S), anti-phospho-AMPK antibody (Thr 172, #2535S) and anti-AMPK (#2532), respectively (all from Cell Signaling). Up to 10^5^ events in the lymphocyte gate were acquired on an LSR-Fortessa (BD) and analyzed using FlowJo 10.0.6 software. Single color controls were included in every experiment for compensation. Isotype controls were also used in every experiment to set up the gates.

### B cell sorting and stimulation of B cell subsets

PBMC were collected using Vacutainer CPT tubes (BD 362761) and cryopreserved. PBMC (1x10^6^/ml) were thawed and cultured in complete medium (c-RPMI, RPMI 1640, supplemented with 10% FCS, 10 μg/ml Pen-Strep, 1mM Sodium Pyruvate, and 2 x 10^−5^ M 2-ME and 2 mM L-glutamine). PBMC were stained with anti-CD19, anti-CD27, anti-IgD antibodies and sorted using a FACS Aria (BD). Cell preparations were typically >98% pure. Naïve B cells (the most frequent B cell subset in blood able to undergo in vivo and in vitro immunoglobulin class switch) or DN B cells were stimulated (10^4^ cells/200 μl) in round-bottom 96-well plates with CpG (2 μg/ml) together with F(ab′)_2_ fragments of goat anti-human IgG (2 μg/ml; Jackson ImmunoResearch Laboratories 109-006-006) for 1–8 days, in the presence of IL-4 (1 μg/ml). F(ab′)_2_ (anti-Ig) stimulation was used to optimally stimulate naïve B cells, as previously shown by us [30] and by others [31]. Proliferation of sorted B cell subsets was measured after 2 day stimulation by ^3^H-Thymidine incorporation.

PBMC were from participants vaccinated during the 2010–2011 and 2011–2012 influenza vaccination seasons, characterized by the same vaccine containing the following viral strains: A/California/7/2009 (H1N1), A/Perth/16/2009 (H3N2), B/Brisbane/60/2008 (B).

For influenza-specific antibody responses, sorted subsets (3-6x10^5^/well) were stimulated with CpG (2 μg/ml) and H1N1 (10 μg/ml) for 10 days, then supernatants collected and measured by ELISA.

For anti-self autoimmune antibody production, the sorted B cell subsets from the same individuals (3-6x10^5^/well) were left unstimulated for 10 days, then supernatants collected and measured by ELISA.

### ELISA to measure antibodies in culture supernatants

H1N1-specific as well as fat-specific IgG in the supernatants of the sorted B cell subsets were measured in stimulated and unstimulated cultures, respectively, by human Ig quantitative ELISA kits (Bethyl Labs E80-104).

To obtain fat antigens, adipocytes from the SAT were centrifuged in a 5415C Eppendorf microfuge (2,000 rpm, 5 min). Total cell lysates were obtained using the M-PER (Mammalian Protein Extraction Reagent, Thermo Scientific), according to the manufacturer’s instructions. Aliquots of the protein extracts were stored at -80°C. Protein content was determined by Bradford [32].

For ds-DNA-specific and Malondealdehyde (MDA)-specific antibody responses we used the following kits: Signosis EA-5002 and MyBioSource MBS390120, respectively.

### Isolation of immune cells from the SAT

SAT was harvested from surgery patients, weighed and washed with 1X Hanks’ Balanced Salt Solution (HBSS). It was then resuspended in Dulbecco’s modified Eagle’s Medium (DMEM), minced into small pieces, passed through a 70 μm filter and digested with collagenase type I (SIGMA C-9263) for 1 hr in a 37°C water bath. Digested cells were passed through a 300 μm filter, and centrifuged at 300 g in order to separate the floating adipocytes (AD) from the stromal vascular fraction (SVF) containing the immune cells. Cell pellet (SVF) on the bottom was resuspended in ACK (Ammonium-Chloride-Potassium) for 3 min at room temperature to lyse the Red Blood Cells. SVF was washed 3 times with DMEM, counted and used for flow cytometry and in vitro experiments.

### PrimeFlow assay

PrimeFlow assay allows the simultaneous measurement of ic mRNA and proteins in specific cells at the single cell level by an amplified Fluorescence In Situ Hybridization technique, in combination with flow cytometry. Briefly, PBMC were left unstimulated, then labeled with Live/Dead detection kit, anti-CD45 and antibodies to detect B cell subsets. For T-bet mRNA and protein detection, after membrane staining, cells were fixed and permeabilized with PrimeFlow RNA buffers, and ic stained with PerCp Cy5.5-conjugated anti-T-bet antibody (Biolegend 644805). Then, target probe hybridization was performed using type 1 (AlexaFluor647) T-bet probe (Affymetrix VA1-1641706). For Sestrin1 and Sestrin 2 mRNA detection, target probe hybridization was performed using type 1 probe for Sestrin 1 (Affymetrix VA1-3008321) and type 6 probe for Sestrin 2 (Affymetrix VA6-3181089). Negative control was the sample without the target probe. Cells were incubated for 2 hrs with the probe in a precisely calibrated incubator set to 40°C. All samples were then incubated with the PreAmplification (PreAmp) reagent for 1.5 hrs and the Amplification (Amp) reagent for an additional 1.5 hrs at 40°C. After signal amplification, cells were incubated with the label probe at 40°C for 1 hr. Cells were washed and suspended in staining buffer prior to acquisition. Approximately 10^5^ events were acquired from each sample on an LSR-Fortessa (BD) and analyzed using FlowJo 10.0.6 software. Spectral compensation was completed using single color control samples. Isotype controls were also used in every experiment to set up the gates.

### RNA extraction and quantitative (q)PCR

After sorting, cells were resuspended in TRIzol (Ambion) (10^6^ cells/100 μl), then RNA extracted for quantitative (q)PCR. Total RNA was isolated according to the manufacturer’s protocol, eluted into 10 μl distilled water and stored at -80°C until use. Reactions were conducted in MicroAmp 96-well plates and run in the ABI 7300 machine. Calculations were made with ABI software. Briefly, we determined the cycle number at which transcripts reached a significant threshold (Ct) for each target gene and for GAPDH as control. A value of the target gene, relative to GAPDH, was calculated and expressed as ΔCt. Reagents and primers (Taqman) were from Life Technologies.

### Glucose uptake measurement

PBMCs (10^6^/mL) were left unstimulated, or they were stimulated with CpG (2 μg/ml) for 3 hrs, at 37°C, 5% CO_2_ and then the fluorescent glucose analog (2-(N-(7-Nitrobenz-2-oxa-1,3-diazol-4-yl)Amino)-2-Deoxyglucose) (2-NBDG, Thermo Fisher N13195) was added to the wells at a final concentration of 50 μM for 30 min. Cells were then washed and stained for 20 min at room temperature with the following fluorochrome-conjugated antibodies: anti-CD45, anti-CD19, anti-IgD, anti-CD27 as well as with the Live/Dead detection kit. Cells were washed and later acquired in a BD LSR Fortessa Flow cytometry instrument, using the FITC channel to detect the signal from the fluorescent glucose uptake tracker. Fluorescence data were analyzed using FlowJo 10.0.6 software.

### Detection of reactive oxygen species (ROS) using CellROX oxidative stress reagent

PBMCs and SVF cells (10^6^/mL) were left unstimulated in c-RPMI at 37°C, 5% CO_2_ for 30 min and then CellROX Deep Red Reagent (Thermo Fisher C10422) was added to the wells at a final concentration of 1 μM. After an additional 30 min incubation, cells were collected and washed with FACS buffer, and stained for 20 min at room temperature with the following fluorochrome-conjugated antibodies: anti-CD45, anti-CD19, anti-IgD, anti-CD27 as well as with the Live/Dead detection kit. Cells were washed and later acquired in a BD LSR Fortessa Flow cytometry instrument, using the APC channel to detect the signal from oxidated CellROX Deep Red Reagent. Fluorescence data were analyzed using FlowJo 10.0.6 software.

### Statistical analyses

Mean comparisons between groups were performed by one-way or two-way ANOVA, or by paired Student’s t test (two-tailed), using GraphPad Prism version 7 software, which was used to construct all graphs.

## Results

### DN B cells do not proliferate to stimuli and do not make antibodies to influenza antigens

**Fig 1:**
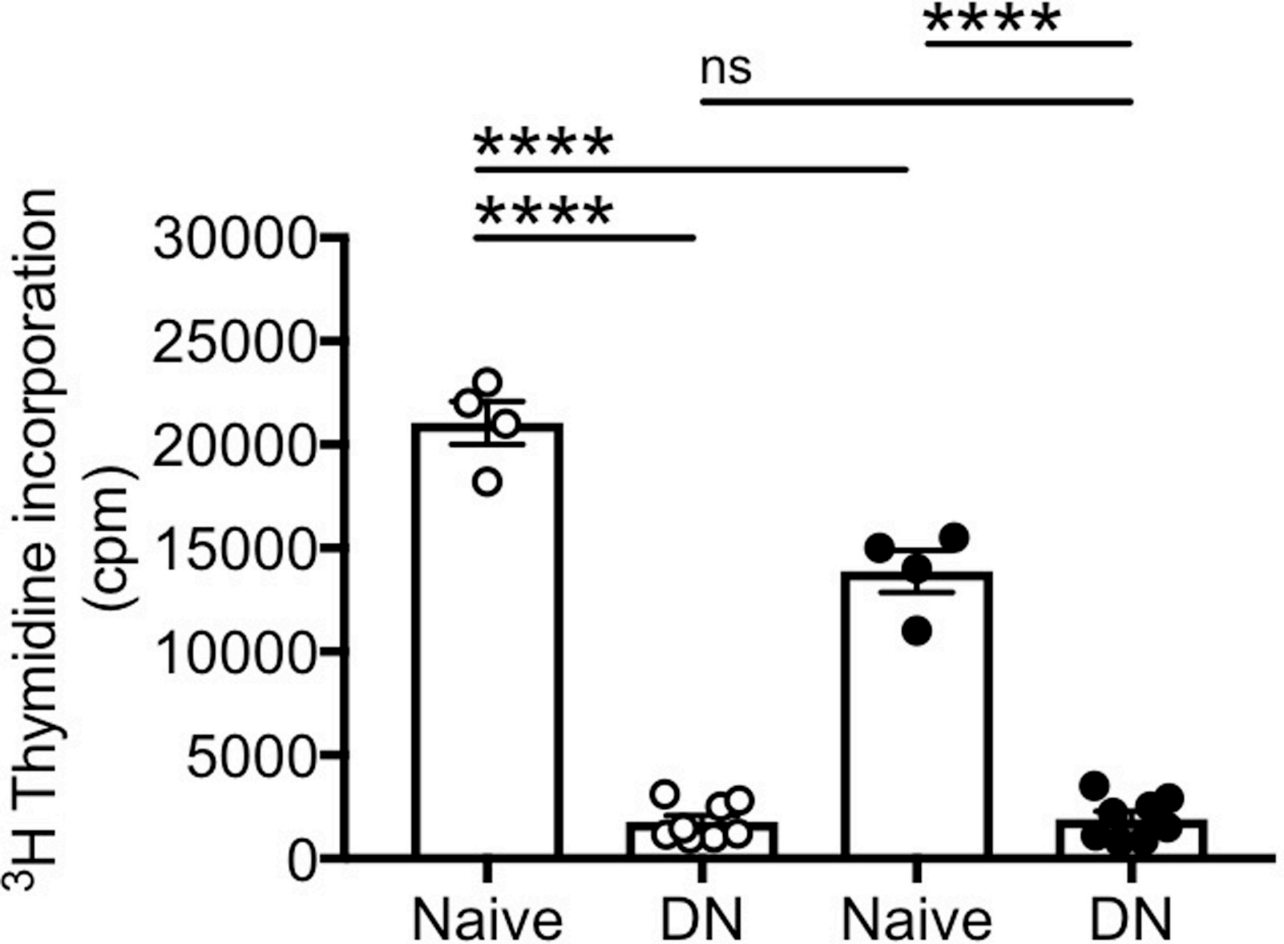
DN B cells do not proliferate. Naïve and DN B cells were sorted from the peripheral blood of 4 young (white symbols) and 8 elderly (black symbols) individuals. Sorted cells were stimulated with CpG and anti-Ig antibodies, together with IL-4. Proliferation of sorted B cell subsets was measured after 2 day stimulation by ^3^H-Thymidine incorporation. Mean comparisons between groups were performed by one-way ANOVA. ****p<0.0001, ns: not significant.

We initially measured cell proliferation of DN B cells, as compared to naïve B cells, as this is the first step in class switch. We measured proliferation by ^3^H-Thymidine incorporation after stimulation with CpG+anti-Ig, together with IL-4, for 48 hrs. Anti-Ig stimulation was used to optimally stimulate naïve B cells, as previously shown by us [30] and by others [31]. Results in Fig 1 show that naïve B cells (which represent the most frequent subset in blood able to undergo in vivo and in vitro immunoglobulin class switch) proliferate well, whereas DN B cells were unable to proliferate or proliferated poorly in response to B cell stimuli. Proliferation was higher in naïve B cells from young as compared to elderly individuals and higher in naïve versus DN in both age groups. Only in a few experiments, we were able to sort IgM memory B cells, due to the low frequency of this B cell subset in blood. IgM memory B cells are the other subset of blood B cells able to undergo in vivo and in vitro class switch. IgM memory B cells proliferated similar to naïve B cells (data not shown).

**Fig 2:**
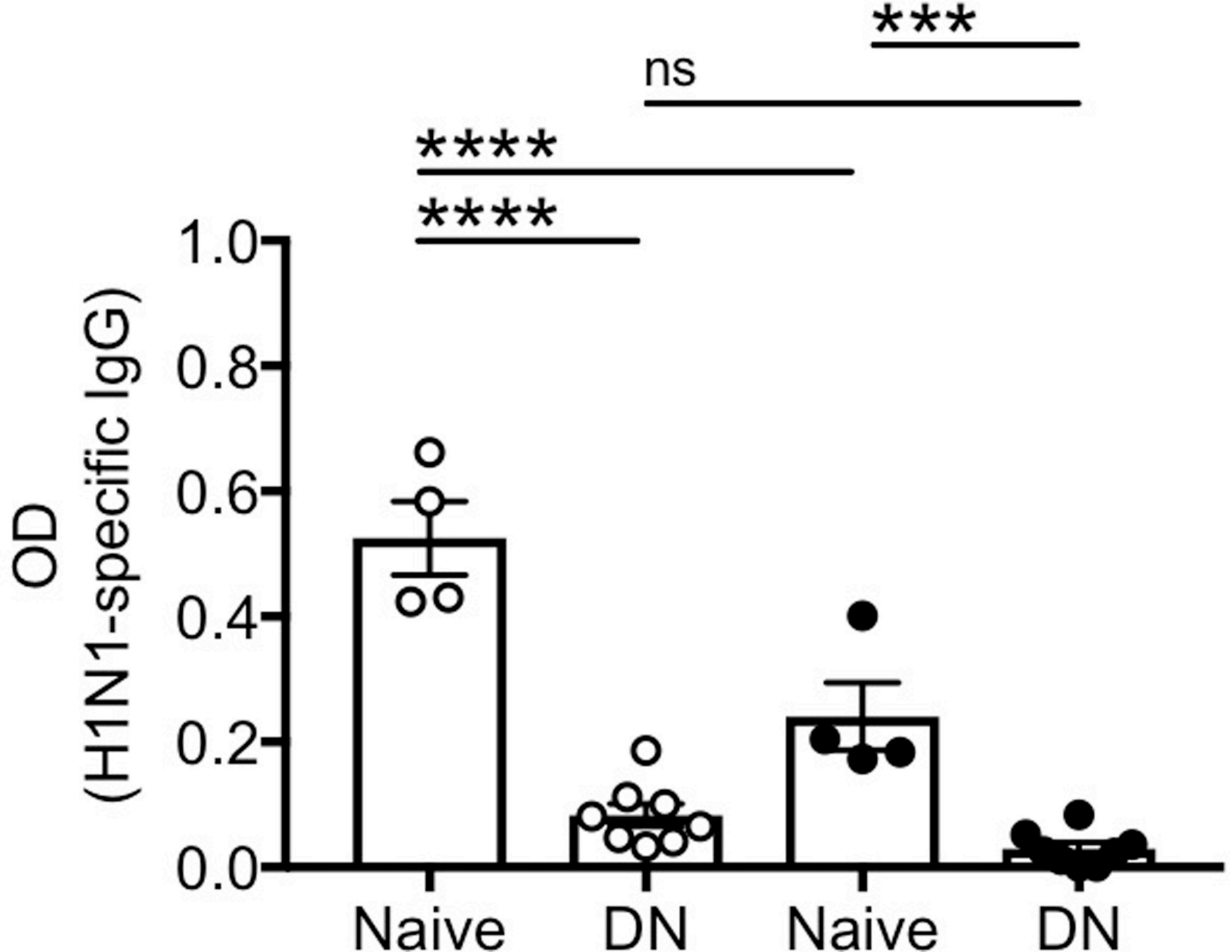
DN B cells do not make influenza-specific antibodies. Naïve and DN B cells were sorted from the peripheral blood of 4 young (white symbols) and 8 elderly (black symbols) individuals. Sorted cells were stimulated with CpG, H1N1 and anti-Ig antibodies for 10 days. Antibody secretion in culture supernatants was evaluated by ELISA using H1N1-coated plates. Mean comparisons between groups were performed by one-way ANOVA. ***p<0.001, ****p<0.0001, ns: not significant.

We then measured the capacity of DN B cells, as compared to naïve B cells, to secrete influenza-specific IgG antibodies after in vitro stimulation/expansion with CpG, H1N1 and anti-Ig antibodies. Influenza-specific responses were measured using H1N1-coated plates by ELISA. These experiments were conducted using PBMC from participants vaccinated during the 2010–2011 and 2011–2012 influenza vaccination seasons, characterized by the same influenza vaccine. Results in Fig 2 show that naïve B cells are able to secrete influenza-specific IgG antibodies, whereas DN B cells are not. These results clearly indicate that DN B cells from vaccinated individuals do not respond to the influenza antigen H1N1. Both IgM memory and switched memory B cells were able to make anti-H1N1 antibodies after in vitro stimulation, in young more than in elderly individuals (data not shown).

Because DN B cells are increased in the blood of healthy elderly individuals [15, 33] and also in the blood of patients with autoimmune diseases [17–22], in which they respond to disease-specific autoantigens, we next tested the hypothesis that these cells may expand in the presence of autoantigens known to increase with age.

### DN B cells spontaneously secrete antibodies with autoimmune reactivity

**Fig 3:**
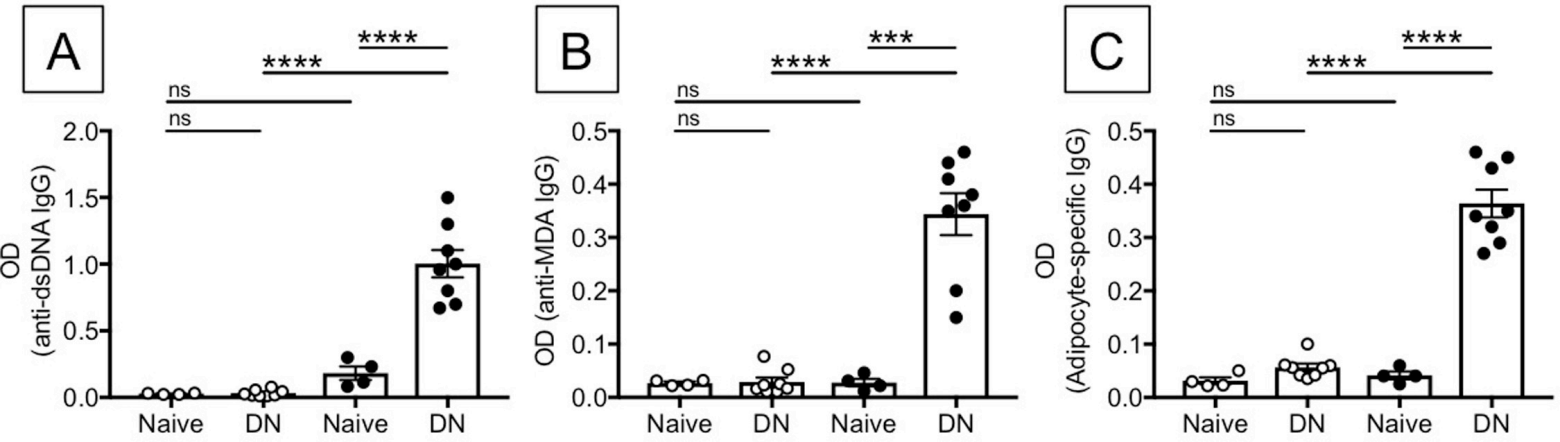
DN B cells make anti-self-specific autoimmune antibodies. Naïve and DN B cells were sorted from the peripheral blood of 4 young (white symbols) and 8 elderly (black symbols) individuals. Sorted cells were left unstimulated for 10 days to evaluate by ELISA the presence of anti-dsDNA antibodies (**A**), anti-MDA antibodies (**B**), and anti-SAT antibodies (**C**) in culture supernatants. Mean comparisons between groups were performed by one-way ANOVA. ***p<0.001, ****p<0.0001, ns: not significant.

We compared the capacity of DN versus naïve B cells to spontaneously secrete antibodies specific for autoantigens known to increase with age, such as the self antigens dsDNA and MDA. We measured MDA as autoimmune antigen because it is a product of lipid peroxidation and a marker of oxidative stress [34], two processes that increase with age. Anti-MDA antibodies are also present in the serum of individuals with autoimmune diseases [35]. We also tested SAT-derived antigens, as fat mass has been shown to increase with age in humans [36]. Results in Fig 3 show that unstimulated DN B cells make autoantibodies with specificity for dsDNA, MDA and SAT-derived antigens, whereas naïve B cells do not. Unstimulated IgM memory and switched memory B cells were unable to secrete autoimmune antibodies of any of the three specificities above (data not shown).

### Unstimulated DN B cells from elderly individuals express high levels of T-bet mRNA

**Fig 4:**
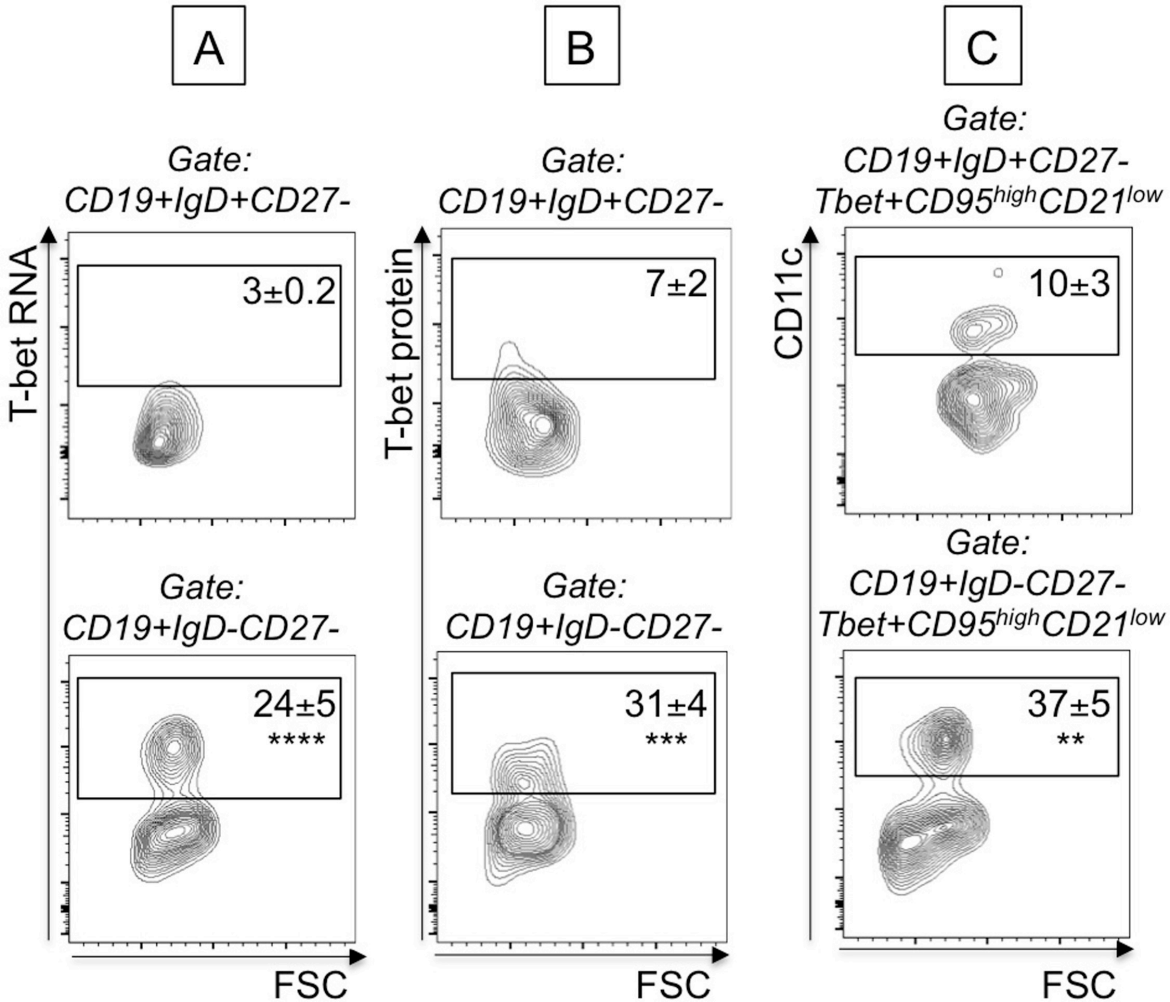
DN B cells from elderly individuals express high levels of T-bet mRNA and protein. B cells from elderly individuals (n = 6) were membrane stained, ic stained to detect T-bet protein and then hybridized with T-bet probe to detect T-bet mRNA. Results show mRNA (**A)** and protein **(B) expression** of T-bet in naïve B cells (CD19+IgD+CD27-, top) versus DN B cells (CD19+IgD-CD27-, bottom). **C.** Results show expression of membrane CD95, CD21 and CD11c markers in naïve B cells (CD19+IgD+CD27-, top) versus DN B cells (CD19+IgD-CD27-, bottom). Events are first gated on naïve/DN B cells, positive for T-bet (mRNA and protein) and then gated on CD95^high^CD21^low^. Mean comparisons between groups were performed by paired Student’s t test (two-tailed). **p<0.01, ***p<0.001, ****p<0.0001.

Secretion of pathogenic (autoimmune) antibodies (IgG2a/c in mice, IgG1 in humans) has been associated with the expression of the transcription factor T-bet [37–39]. We therefore measured T-bet expression in B cell subsets from elderly individuals, as only DN from the elderly were secreting antibodies with anti-self specificity. We used the PrimeFlow RNA assay, in the absence of any stimulation. This technique allows measurement of mRNA in specific subsets of B cells by an amplified, Fluorescence In Situ Hybridization technique, in combination with flow cytometry and therefore allows the simultaneous evaluation of T-bet mRNA and protein expression in B cell subsets. Briefly, B cells were membrane stained with anti-CD19/CD27/IgD to identify subsets, then ic stained with anti-T-bet antibody and then hybridized with T-bet mRNA probe. Results in Fig 4 show that T-bet mRNA and protein expression are higher in unstimulated DN versus naïve B cells, suggesting that in the presence of chronic exposure to “self” antigens T-bet may drive secretion of autoimmune antibodies. DN B cells spontaneously expressing T-bet are CD95+CD21-CD11c+, a phenotype associated with autoimmunity. The frequency of T-bet+CD95+CD21-CD11c+ were found higher in DN versus naïve B cells. These results indicate that the subset of DN B cells is already pre-activated and this is why the expression of immune activation markers is higher as compared to the other B cell subsets, as we have previously shown [15].

### Obesity accelerates age defects in B cells and is associated with higher frequencies of DN B cells

**Fig 5:**
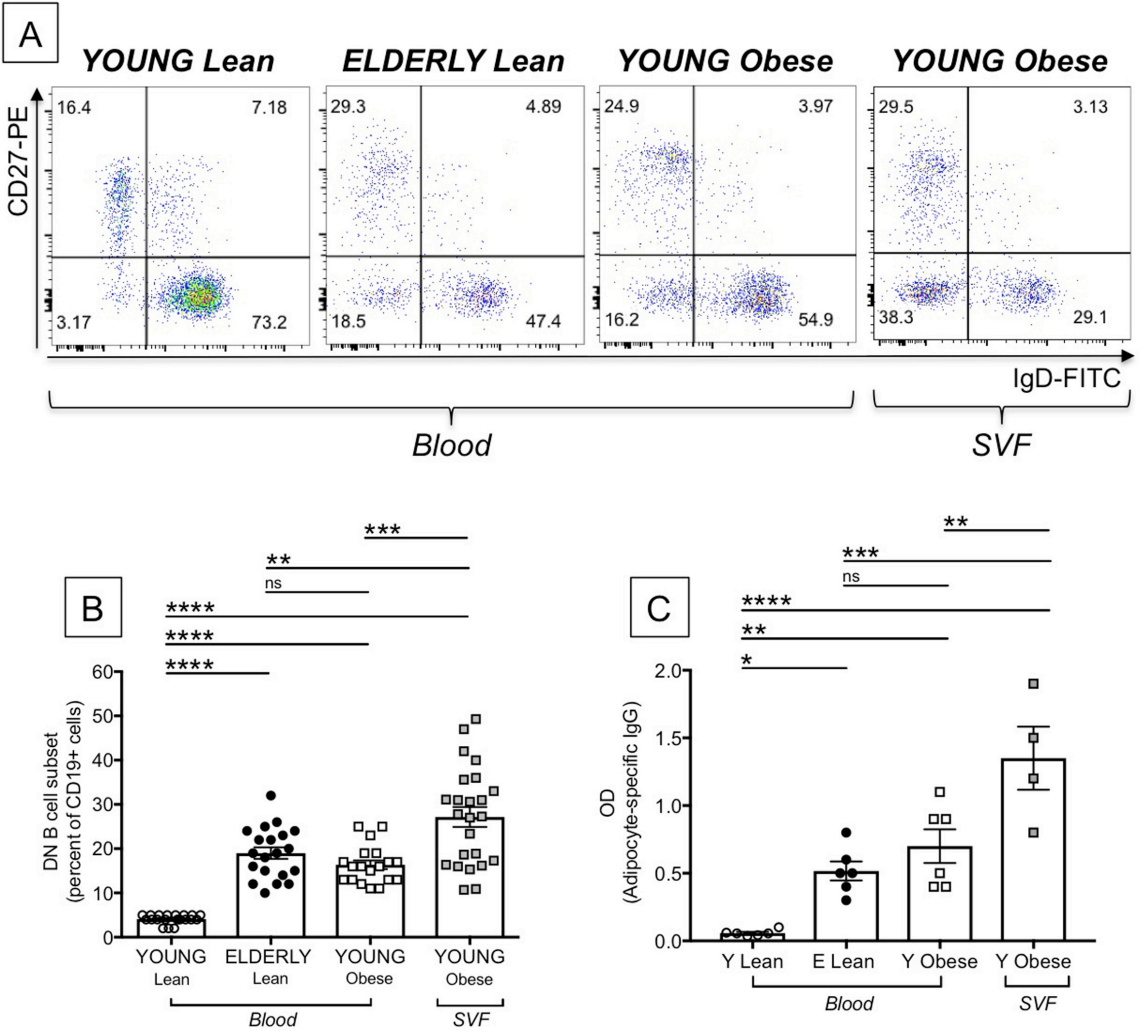
Effects of aging and obesity on the frequency and function of DN B cells. PBMC from the blood from young and elderly lean individuals and from the blood of young obese individuals were stained to evaluate the frequencies of DN B cells. The SVF from the SAT of young obese individuals was also stained. Young individuals giving PBMC and the SAT were age-, gender- and BMI-matched. **A.** Representative dot plots are shown. **B.** Results are expressed as percentages of CD19+ B cells. **C.** Sorted B cell subsets were left unstimulated in culture for 10 days, then supernatants were collected and tested in ELISA for the presence of IgG specific for protein lysates from the SAT. Mean comparisons between groups were performed by one-way ANOVA. **p<0.01, ***p<0.001, ****p<0.0001, ns: not significant.

Obesity is an inflammatory condition that induces defects in B cells similar to those induced by aging [40]. Obesity is also associated with increased frequencies of DN not only in blood [40] but also in the AT as we have recently shown for the obese SAT [41]. Here we compare the frequencies of DN B cells in the blood of young and elderly lean individuals, in the blood and the SVF from the SAT of young obese individuals. Young individuals giving PBMC and the SAT were age-, gender- and BMI-matched. Results in Fig 5A show representative dot plots of the major B cell subsets in the blood of the four groups of individuals. Fig 5B shows that DN B cells are significantly increased in the blood of elderly individuals, as we have previously reported [15, 33]. The blood of obese young individuals is also significantly enriched in DN B cells, confirming our initial observations that obesity like aging induces higher percentages of DN B cells as compared to lean controls. In the SVF from the SAT, DN frequencies are even higher and in some individuals reach 50% of the B cell pool.

We then compared the capacity of DN B cells from the blood of young and elderly lean individuals, as well as from the blood of individuals with obesity and from the obese SAT, to secrete antibodies with anti-“self” specificity. Only a few experiments were performed with DN from the SVF due to the difficulties we experienced sorting cells from samples with high content of lipid droplets. Results in Fig 5C show that DN B cells from the SAT secreted the highest amount of fat-specific antibodies, as compared to DN B cells from the blood. These results are to our knowledge the first to show that the human obese SAT is enriched in antibodies with specificity for “self” antigens, secreted by the subset of DN B cells as a consequence of high mortality occurring in the expanding SAT, leading to the release of “self” antigens, as we have previously demonstrated [41].

### Metabolic requirements of DN B cells in aging and obesity

Effector function is intrinsically linked to cell metabolism. Studies conducted on mouse and human B cells have shown that metabolic reprogramming is required for class switch and antibody production after mitogen or antigen stimulation [42]. However, nothing is known about metabolic requirements of mouse and human B cell subsets and especially of unstimulated B cell subsets. DN B cells rely on metabolic reprogramming for effector function (autoantibody production) and survival. This metabolic reprogramming involves increased expression of glycolytic and mitochondrial components. We compared glucose uptake and RNA levels of enzymes involved in glycolysis and fatty acid oxidation in DN B cells from the blood of young and elderly lean individuals, as well as from the blood of individuals with obesity and from the obese SAT.

**Fig 6:**
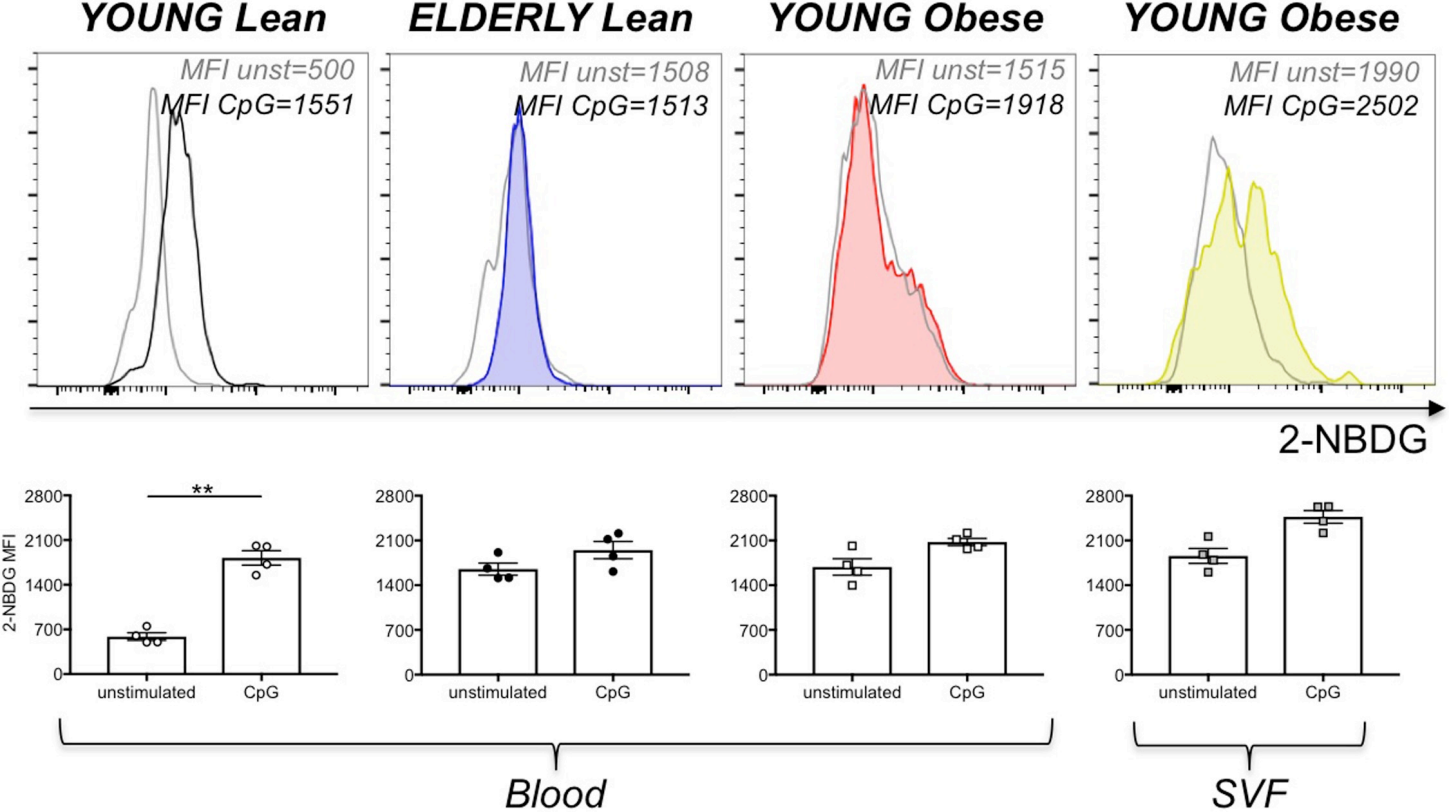
Effects of aging and obesity on glucose uptake in DN B cells. PBMC from the blood from young and elderly lean individuals and from the blood of young obese individuals were stained to evaluate the frequencies of DN B cells. The SVF from the SAT of young obese individuals was also stained. Young individuals giving PBMC and the SAT were age-, gender- and BMI-matched. Cells were incubated in the presence of 2-NBDG. **Top**. Representative histograms of 2-NBDG uptake are shown for each group of individuals. **Bottom**. Mean Fluorescence Intensity (MFI)±SE of 2-NBDG uptake before and after CpG stimulation. Mean comparisons between groups were performed by Student’s t test (two-tailed). **p<0.01.

We initially measured glucose uptake in unstimulated and stimulated DN B cells by using flow cytometry and the glucose fluorescent analog 2-NBDG. Young individuals giving PBMC and the SAT were age-, gender- and BMI-matched. Results in Fig 6 show increased glucose uptake in unstimulated DN B cells from elderly and obese versus young individuals and more in the SVF from the SAT as compared to the blood. Stimulation with CpG leads to increased glucose uptake only in DN B cells from young lean individuals, suggesting that DN from lean elderly [15] and obese young individuals [40, 41] are already pre-activated and able to secrete antibodies with anti-“self” reactivity without additional stimulation.

**Fig 7:**
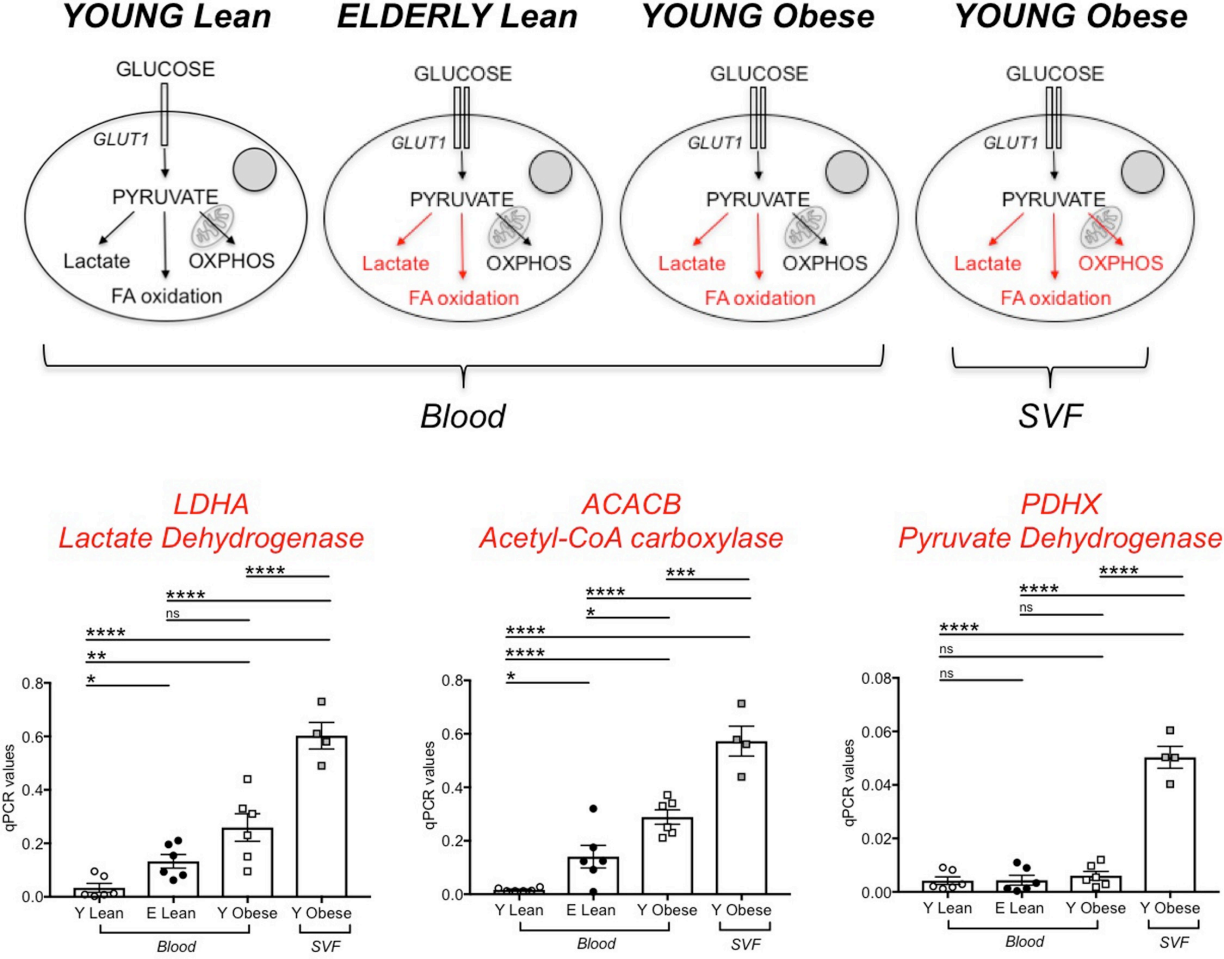
Effects of aging and obesity on glycolytic measures in DN B cells. PBMC and SVF were obtained as indicated previously. **Top**. A model to explain the different glycolytic pathways in the different groups is shown. In red are indicated activated pathways. **Bottom**. Results show qPCR values (2^-ΔΔCt^) of LDHA, ACACB and PDHX. Mean comparisons between groups were performed by one-way ANOVA. *p<0.05, **p<0.01, ***p<0.001, ****p<0.0001, ns: not significant.

We next measured RNA levels of enzymes involved in glycolysis and fatty acid oxidation in unstimulated DN B cells from the blood of young and elderly lean individuals, as well as from the blood of individuals with obesity and from the obese SAT. Again, only a few experiments were performed with DN from the SVF due to the difficulties we experienced sorting cells from samples with high content of lipid droplets. We measured in particular LDHA (lactate dehydrogenase) that converts pyruvate into lactate and represents a measure of aerobic glycolysis; ACACB, Acetyl-CoA carboxylase, a regulator of fatty acid synthesis; PDHX, a component of the pyruvate dehydrogenase complex that converts pyruvate into acetyl-CoA and represents a measure of oxidative phosphorylation (OXPHOS) and mitochondrial function. Results in Fig 7 show that DN B cells from lean young individuals have minimal metabolic requirements, as they need glucose to generate just the amount of ATP necessary to survive. DN B cells from lean elderly individuals and from obese young individuals utilize higher amounts of glucose to perform autoimmune antibody production and enroll in aerobic glycolysis to support their function. DN B cells in the SVF from the SAT have the highest metabolic requirements and they activate oxidative phosphorylation, aerobic glycolysis and fatty acid oxidation.

**Fig 8:**
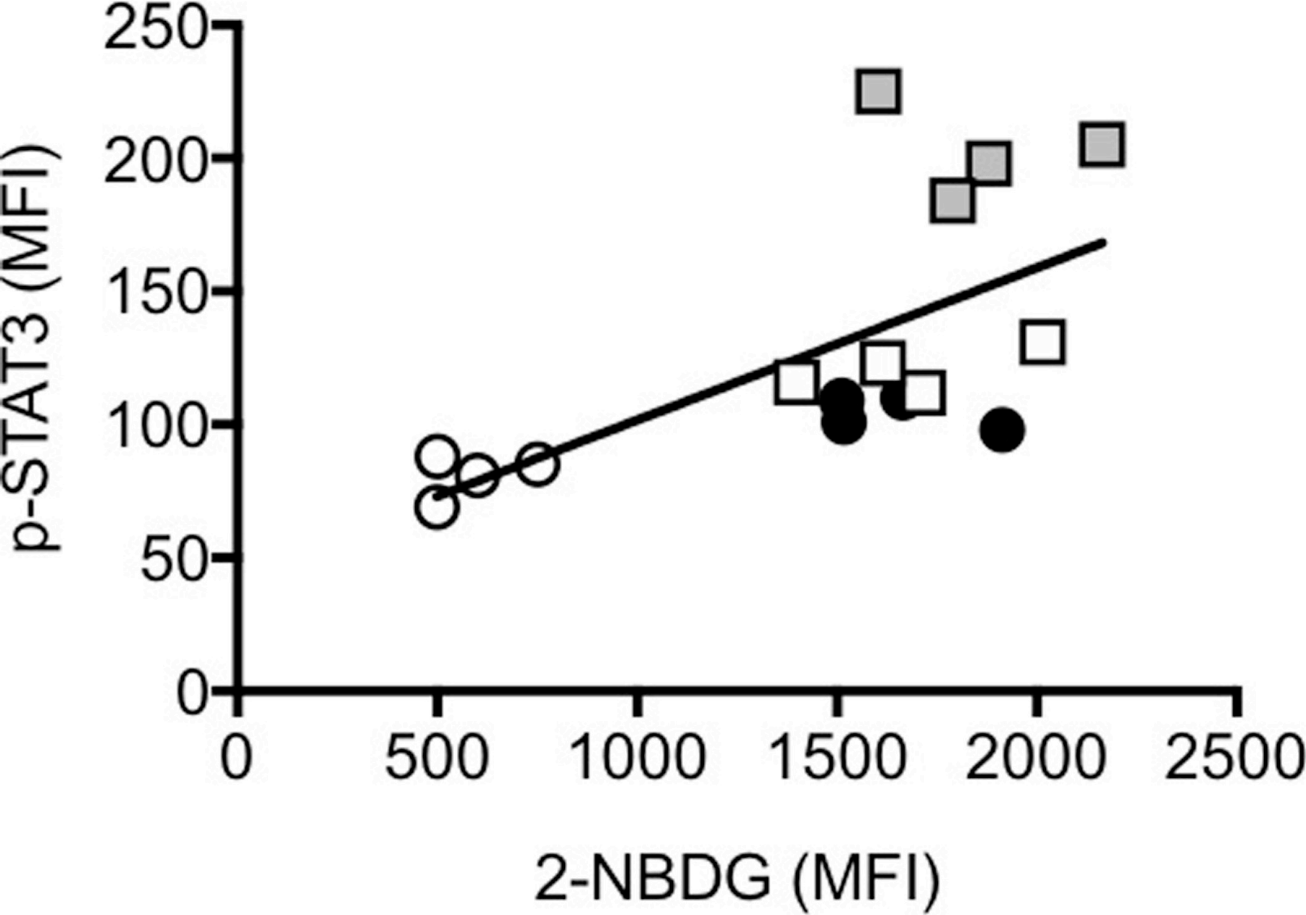
Glucose uptake is positively associated with ic phospho-STAT3 in DN B cells. PBMC and SVF were obtained as indicated previously. Glucose uptake and ic phosphor-STAT3 were measured in DN B cells from the same individuals. Pearson’s correlation r = -0.65, p = 0.007. Symbols are as follows: young lean, white circles; elderly lean: black circles; young obese: white squares; young obese SVF: grey squares.

Glucose is a critical component of the pro-inflammatory response of macrophages and hyperglycaemia has been shown to induce phosphorylation of STAT3 and secretion of pro-inflammatory cytokines in mouse peritoneal macrophages [43]. Results in Fig 8 show that DN B cells with increased glucose uptake (measured by 2-NBDG) also have spontaneous phosphorylation of STAT3 (measured by ic phosphorylated STAT3), with the highest levels observed in SAT-derived DN B cells. Glucose uptake and ic phospho-STAT3 were measured in DN B cells from the same individuals. The reason to measure STAT3 is because we have experimental evidence that peripheral blood B cells (total CD19+), stimulated with glucose (3–30 mM) spontaneously phosphorylate STAT3 and express mRNA for the pro-inflammatory cytokines TNF-α and IL-6 in a dose-dependent manner. In the absence of glucose ther is no phosphorylation of STAT3 (data not shown). STAT3 binds several NF-kB factors inducing its translocation from the cytoplasm to the nucleus and its constitutive activation, leading to the transcription of genes for pro-inflammatory cytokines [44].

**Fig 9:**
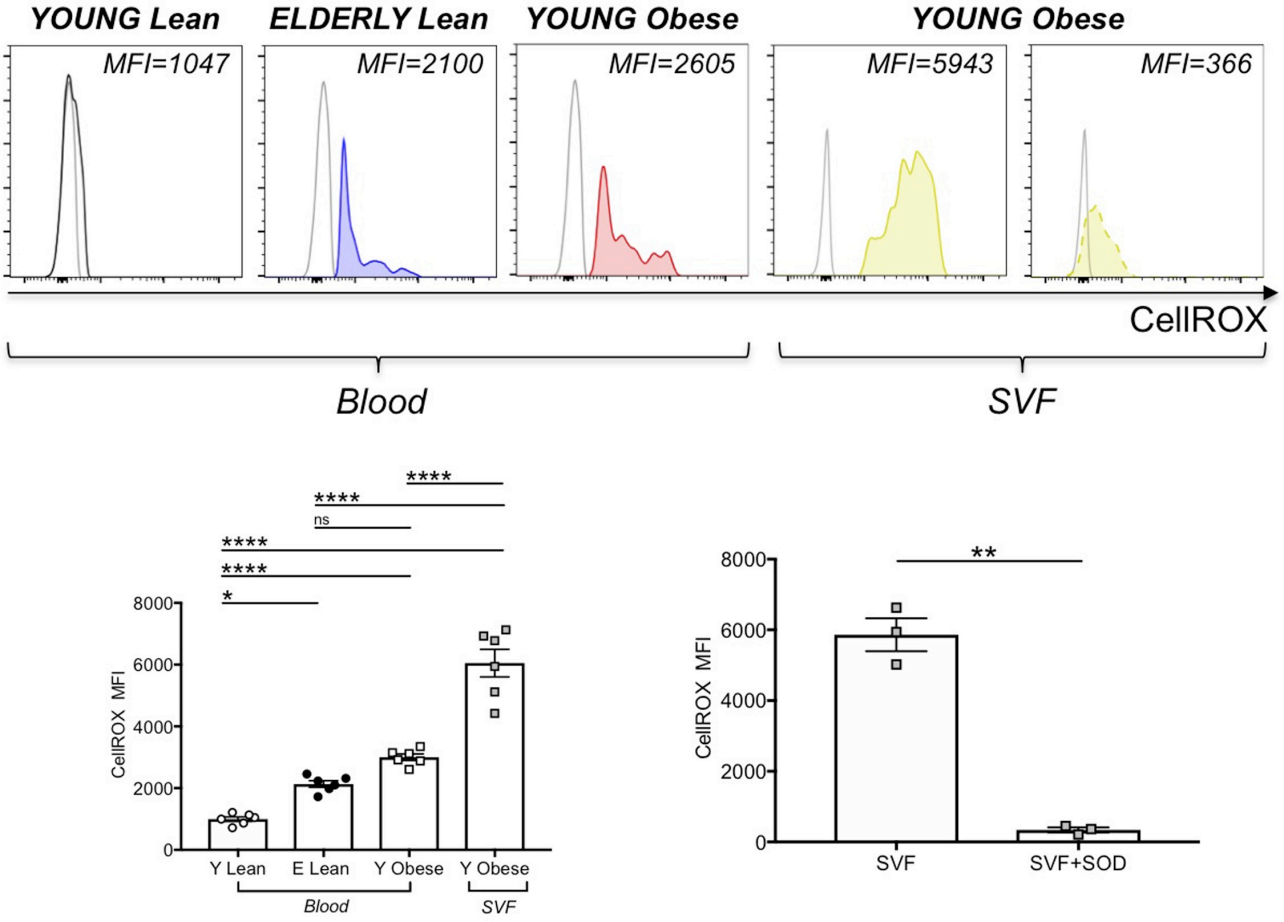
Effects of aging and obesity on ROS in DN B cells. PBMC and SVF were obtained as indicated previously. **Top**. Representative histograms of CellROX are shown for each group of individuals (solid lines). The dotted line in the last histogram indicates CellROX staining in SVF samples pre-incubated with Vitamin E (100 μg/10^6^ SVF). **Bottom, left**. Mean Fluorescence Intensity (MFI)±SE of CellROX. In each histogram, unstained cells are used as negative controls. Mean comparisons between groups were performed by one-way ANOVA. *p<0.05, ****p<0.0001, ns: not significant. **Bottom, right**. The SVFs from 3 young obese individuals (among those in the left graph) were pre-incubated for 30 min with Vitamin E before adding CellROX. Results show MFI±SE of CellROX. Mean comparisons between groups were performed by Student’s t test (two-tailed). **p<0.01.

We then evaluated if the metabolic reprogrammimg of DN B cells in aging and obesity was accompanied by mitochondrial changes in mass and function. We measured mitochondrial mass by flow cytometry and the green fluorescent mitochondrial stain Mitotracker Green, before and after stimulation with CpG, and we found no differences in mitochondrial mass when we compared DN B cells from young lean, elderly lean and young obese individuals, either before or after stimulation (data not shown). When we measured the expression of ROS in unstimulated DN B cells, using the deep red fluorogenic probe CellROX, we found higher levels of ROS in DN B cells from the blood of elderly lean and young obese individuals, as compared to young lean individuals. The highest levels of expression were found in DN B cells from the SAT (Fig 9). To validate the specificity of the staining with CellROX, the SVFs from 3 young obese individuals were pre-incubated for 30 min with an antioxidant compound before the addition of the CellROX reagent. We used α-tocopherol (Vitamin E), a potent lipid-soluble antioxidant, because it is a scavenger of reactive oxygen species able to reduce oxidative stress and inflammation in immune cells, as reviewed in [45]. Pre-incubation, as expected, significantly reduced the oxidation of CellROX. These results are in agreement with the hypothesis that higher glucose utilization induces a ROS-driven pro-inflammatory phenotype in DN B cells, as already shown in macrophages [46].

### Sestrins and AMPK activation in DN B cells in aging and obesity

**Fig 10:**
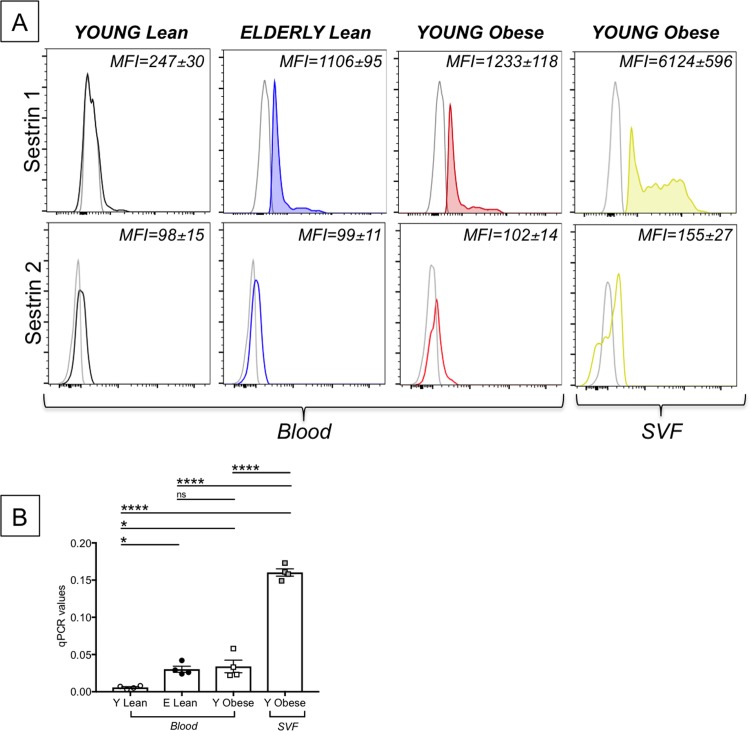
Effects of aging and obesity on Sestrin 1 and Sestrin 2. PBMC and SVF were obtained as indicated previously (n = 4). **A.** Results show mRNA expression of Sestrin 1 (**top**) and Sestrin 2 (**bottom**), both measured by PrimeFlow. **B.** qPCR values (2^-ΔΔCt^) of Sestrin 1. Mean comparisons between groups were performed by two-way ANOVA. *p<0.05, ****p<0.0001, ns: not significant.

AMPK (5’-AMP activated kinase) has been shown to regulate energy metabolic homeostasis and cell survival during stress. Sestrins, upstream of AMPK, also promote cell survival under stress conditions and regulate AMPK activity. We first measured the expression of Sestrin 1 and Sestrin 2 in unstimulated DN B cells. Results in Fig 10A (top) show that the expression of Sestrin1 mRNA was higher in DN B cells from the blood of elderly lean and young obese individuals, as compared to young lean individuals, with the highest levels of expression in DN B cells from the SAT. Levels of Sestrin 2 mRNA, conversely, were comparable in the four groups (Fig 10A, bottom). The up-regulation of Sestrin 1 observed by PrimeFlow was confirmed by qPCR (Fig 10B).

**Fig 11:**
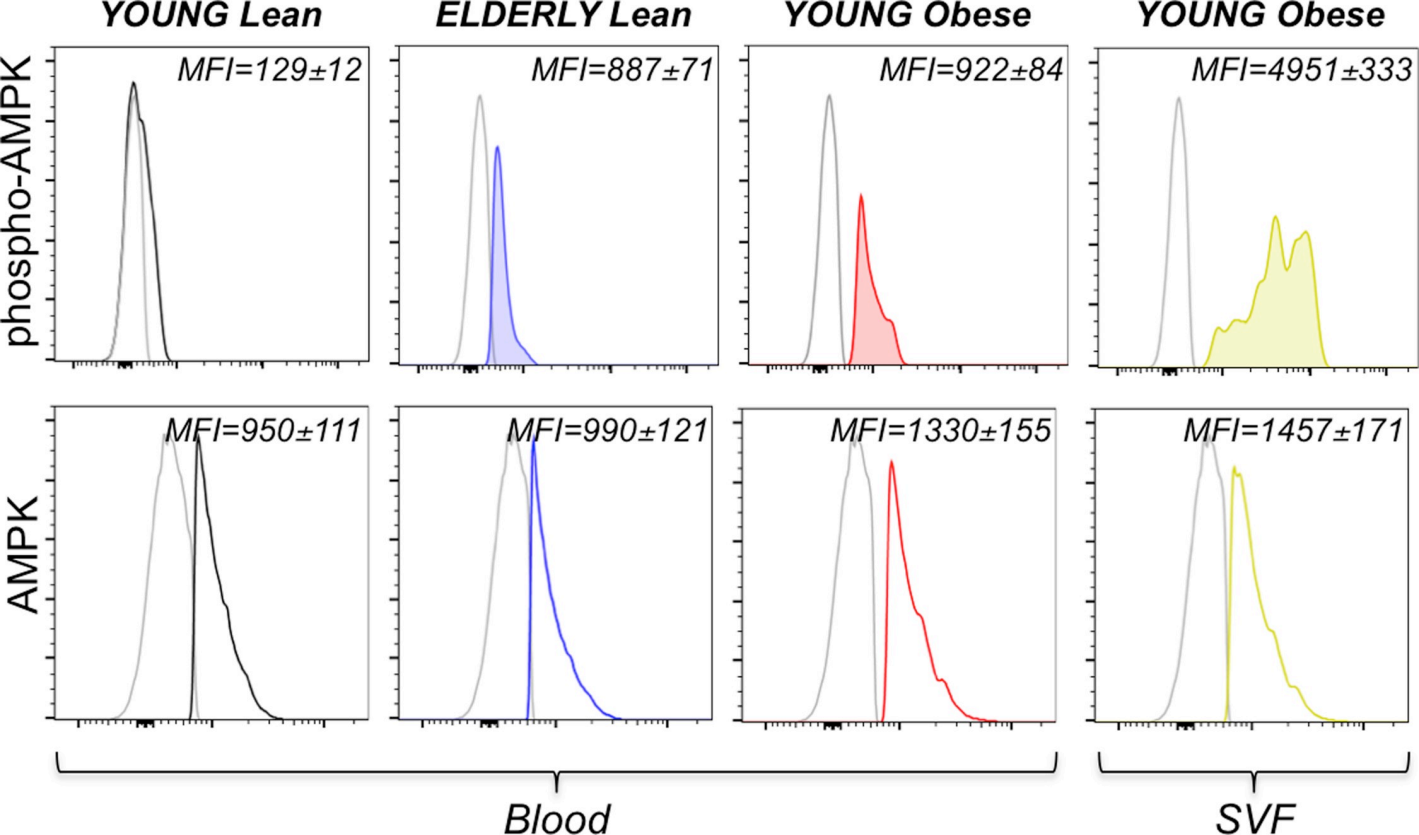
Effects of aging and obesity on AMPK. PBMC and SVF were from the same 4 individuals in Fig 10. Results show phospho-AMPK (**top**) and total AMPK (**bottom**), both measured by ic staining. Mean Fluorescence Intensity (MFI)±SE is shown in each quadrant.

We then measured the expression of phosphorylated and total AMPK in the same individuals in Fig 10. Results in Fig 11 show that phospho-AMPK was also higher in DN B cells from the blood of elderly lean and young obese individuals, as compared to young lean individuals. The highest levels of phospho-AMPK expression were observed in DN B cells from the SAT. Levels of total AMPK were comparable in the four groups. These results altogether clearly indicate that DN B cells activate pathways that mitigate stress and cell death and this gives them a metabolic advantage based on their ability to optimize nutrient usage. This metabolic advantage supports their survival and function in a hostile highly inflammatory milieu.

## Discussion

Results presented herein show that DN B cells accumulate in the blood of elderly individuals, do not proliferate to stimuli and do not make antibodies to influenza antigens. However, they secrete antibodies with autoimmune specificity. We have tested anti-dsDNA and MDA specificities as well as specifities for proteins present in lysates from the SAT, and found antibodies against these specifities higher in DN as compared to the other B cell subsets isolated from the blood of elderly individuals. Our results are to our knowledge the first to show that DN from elderly individuals have specificities for fat-associated antigens and this finding, although not previously shown, is not surprising, as fat mass increases with age in humans [36]. It is likely that the mechanisms for the release of fat-associated self antigens in the AT of elderly individuals are similar to those we have described in the obese SAT, leading to the secretion of autoimmune antibodies.

DN B cells also accumulate in the blood of individuals with obesity and more in the SVF from the SAT as compared to the blood. DN B cells from the SVF of the SAT are responsible for the secretion of antibodies with anti-“self” specificity in the obese SAT. They do so because they perform a metabolic adaptation which gives them a metabolic advantage.

It is well known that rapid metabolic changes are needed by B cells to transition from a naïve to a memory/activated phenotype and this occurs through increased glucose consumption, increased glycolysis, increased mitochondrial mass and dysfunction. In particular, it has been shown that mitogen- or antigen-stimulated B cells increase their glycolysis rate, expression of the glucose transporter GLUT1 through mechanisms dependent on c-Myc and PI3K, and increased OXPHOS [42, 47]. In the highly inflammatory environment of the blood of elderly individuals and individuals with obesity, and even more in the SVF from the obese SAT, however, all these pathways are already activated in unstimulated DN B cells, and at higher extent as compared to the other unstimulated B cell subsets, leading to their constitutive activation and secretion of autoimmune antibodies which have been described to be pathogenic [48].

Metabolic adaptation occurs after activation of pathways that mitigate stress and cell death, leading DN B cells to better optimize nutrient usage. This metabolic advantage drives their survival in a hostile milieu, allowing these cells to perform their function and secrete autoimmune antibodies. Here we have evaluated Sestrin 1 and AMPK. Sestrin 1 is a member of the family of evolutionarily-conserved Sestrins whose expression is upregulated in cells exposed to environmental stresses such as DNA damage, oxidative stress and hypoxia [49]. Sestrin 1 has been shown to activate AMPK and therefore is a crucial regulator of metabolic homeostasis. Sestrin-induced AMPK activation has also been shown to induce peroxisome proliferator-activated receptor γ (PPARγ) [50, 51], resulting in increased mitochondrial biogenesis, mitophagy, removal of dysfunctional mitochondria, decreased intracellular accumulation of ROS and activation of anti-oxidant defenses. Sestrin-induced AMPK also inhibits mTORC1 [52, 53] and NADPH Oxidase 4, both responsible for the generation of pathogenic amounts of ROS [54]. These results altogether highlight the impact of stress sensing pathways on DN B cell survival and also highlight possible mechanisms enabling them to make autoantibodies. Further understanding of the regulation of these metabolic pathways will provide new directions to suppress autoantibody production during aging, obesity and other inflammatory-based conditions.
